# Assessment of drug-related problems at the emergency department in older patients living with frailty: pharmacist-led medication reviews within a geriatric care team

**DOI:** 10.1186/s12877-023-03942-x

**Published:** 2023-04-05

**Authors:** Merel van Nuland, Madelon Butterhoff, Karin Verwijmeren, Florine Berger, Vera M. Hogervorst, Annemarieke de Jonghe, Paul D van der Linden

**Affiliations:** 1 Department of Clinical Pharmacy, Tergooi Medical Center, Van Riebeeckweg 212, 1213 XZ Hilversum, the Netherlands; 2 Department of Geriatrics, Tergooi MC, Hilversum, the Netherlands

**Keywords:** Elderly, Frailty, Medication review, Drug-related problem (DRP), Emergency department

## Abstract

**Background:**

Older patients are vulnerable to experiencing drug related problems (DRPs), which may result in emergency department (ED) visits. However, it is not standard practice to conduct medications reviews during ED visit. The aim of this study was to assess the number of DRPs in older patients living with frailty at the ED, identified through pharmacist-led medication reviews within a geriatric care team, and to determine the acceptance rate of pharmacists’ recommendations among hospital physicians and general practitioners or elderly care specialists.

**Methods:**

A retrospective observational study was performed in patients ≥ 70 years living with frailty at the ED at Tergooi Medical Center. Pharmacist-led medication reviews were conducted to identify and classify DRPs as part of a larger geriatric assessment. The acceptance rate of given recommendations was determined during follow-up.

**Results:**

A total of 356 ED visits were included. The mean (standard deviation, SD) age of patients was 83 (6.8) years. About 76% of patients had at least one DRP. In total, 548 DRPs were identified with a mean of 1.5 DRP (SD 1.3) per patient. The acceptance rate of medication recommendations in admitted patients was 55%, and 32% among general practitioners/elderly care specialists in discharged patients.

**Conclusions:**

Pharmacist-led medication reviews as part of a geriatric care team identified DRPs in 76% of older patients living with frailty at the ED. The acceptance rate was substantially higher in admitted patients compared to discharged patients.

**Supplementary Information:**

The online version contains supplementary material available at 10.1186/s12877-023-03942-x.

## Background

Adverse drug events are a significant contributor of emergency department (ED) visits [1]. Older patients are particularly susceptible to these events, as well as hospital visits and admissions [2]. This vulnerability may be attributed to various factors such as multi-morbidities, polypharmacy, and declines in cognitive and functional abilities which can impact correct drug usage [2]. Furthermore, the risk of hospital-associated disability, defined as a decline in independence in daily activities following hospitalization, is higher among older patients and negatively impacts their quality of life after discharge [3]. Approximately 10% of unscheduled ED visits among older patients are related to drugs, with 5% of these events being potentially avoidable [4]. Drug-related problems (DRPs) not only lead to increased healthcare costs and hospital admissions, but also represent a critical issue that needs to be addressed, particularly in the elderly population [5].

There are several strategies for identifying DRPs in admitted patients, [6] including medication review programs [6]. Medication review involves a systematic and thorough examination of a patient's medications with the goal of improving health outcomes [7]. Previous studies have demonstrated the impact of in-hospital medication reviews on readmission rates, length of hospital stay, and mortality. Although its impact on mortality was limited [5, 8, 9], other studies have indicated shorter hospital stays, reduced ED visits, heightened patient satisfaction, and decreased healthcare costs [5, 10–11]. Additionally, pharmacist interventions in the ED was linked to more accurate and thorough medication histories, and higher prescription appropriateness [12]. While the effects of in-hospital medication reviews have been well-researched, the impact of medication reviews for older patients living with frailty in the ED has yet to be fully studied. This is despite the high number of drug-related ED visits and the inherent risks associated with the transition from emergency care to hospital treatment. This may be due to the challenges posed by the fast-paced and hectic environment of the ED, as well as the limited duration of the patient's stay. Given the heightened susceptibility of older patients in the ED to adverse events, it is believed that ED medication reviews could be beneficial for this population.

The Dutch multidisciplinary guideline on polypharmacy in older patients (aged 70 years and older) and the Dutch geriatric guideline recommend medication reviews for older patients with polypharmacy (taking at least 5 medications) who present at the ED [13]. In line with these guidelines, pharmacists at Tergooi Medical Center (MC) initiated medication reviews for older patients living with frailty (aged 70 years and older) in the ED as part of a larger geriatric care team, starting in May 2020. In this retrospective observational study, results of these medication reviews were analyzed. The aim of this study was to assess the number of DRPs in older patients living with frailty at the ED, identified through pharmacist-led medication reviews within a geriatric care team, and to determine the acceptance rate of pharmacists’ recommendations among hospital physicians and general practitioners (GPs) or elderly care specialists. The novelty of this study lies in the integration of pharmacists as members of the geriatric care team, the study population being older patients living with frailty, and the distinctive setting of the ED.

## Methods

### Geriatric assessment

A retrospective observational study was performed at the ED of the Tergooi MC, Hilversum, The Netherlands. Tergooi MC is a secondary care teaching hospital with 322 hospital beds. Pharmacist-led medication reviews were performed by a geriatric care team in routine care during working hours (08:30–17:00). Older patients (≥ 70 years) visiting the ED were screened for frailty by the ED nurse. Frailty was determined by a questionnaire (Table 1) which consisted of four questions [14]. Besides, the ED nurse could also indicate, based on her clinical experience, the patient to be frail. When a positive response was recorded to any of the questions, the patient was classified as frail.

**Table 1:** Questionnaire for determining clinical frailty in older patients at the ED

Questions for determining clinical frailty
Does the patient have memory problems?
Is the patient confused/delirious or did the patient experience a previous delirium?
Is a fall the reason for presentation at the emergency department?
Does the patient require more care in the home-situation or admission to a nursing home if the patient is not admitted to the hospital?

Frail patients were seen by an advanced nurse practitioner (ANP) or physician of the geriatric department. Thereafter, medication reconciliation was performed by the pharmacist technician to obtain a complete list of the patient’s current drugs. The procedure involved the following steps: (1) obtainment of the most recent drug profile from the public pharmacy, (2) conducting an interview with the patient to ascertain their current drugs, (3) compilation of the final drug profile using the information obtained from the interview and pharmacy records. This drug profile served as the basis for the pharmacist-led medication review. Pharmacists participating in the study were either registered hospital pharmacists or residents. A brief training was provided by the project initiator, who was a hospital pharmacist specializing in geriatric care. DRPs identified through the medication review were evaluated by the pharmacist and the ANP or a geriatrician. During this process, recommendations were given for each DRP. The type of recommendation was directly related to the type of DRP. For instance, in the case of an excessive dose, the recommendation was to reduce the dose. These recommendations were then communicated to the treating physician by the ANP or geriatrician by telephone and were documented in the electronic health record (EHR) system.

For patients discharged directly from the ED, the medication recommendations were transmitted to the GP or elderly care specialist through a discharge letter [15, 13–16]. The discharge letter was composed by the ANP or geriatrician, and recommendations documented in the EHR were incorporated in this letter. To monitor the follow-up of recommended interventions, the patients' GP or elderly care specialists were contacted by a pharmacist via mail within six months after their ED visit. In the event of non-response, the physician was contacted via telephone.

### Medication review and definition of DRPs

Medication reviews were performed by a pharmacist to identify DRPs. DRPs were defined as any undesirable event with drug therapy that interferes or has the potential to interfere with desired health outcomes. This includes adverse drug events, adverse drug reactions and medication errors [17, 18].

Several references were used to identify DRPs. First, the trigger tool (Supplementary Table 1) was used to identify adverse events in relation to the reason of presentation at the ED [4, 13]. Second, the screening Tool to Alert to Right Treatment (START)- and Screening Tool of Older Persons' Prescriptions (STOPP)-criteria were used [19]. Third, Ephor guidelines were consulted for dose recommendations in older patients; Ephor is an expert center for pharmacotherapy in older patients [16]. Last, multidisciplinary guidelines were used in the medication review for identifying DRPs. The mean time for performing a medication review was recorded.

### Study population

Patients ≥ 70 years or older who visited the ED during the study period (7 May 2020 to 31 December 2020) and positively screened for frailty were included. Critically ill patients and patients with a suicide attempt were excluded.

### Data collection

The reason for ED visit, as documented by the treating physician, was collected from the EHR. Furthermore, DRPs and recommendations were collected from written notes by the pharmacists in the EHR. The Pharmaceutical Care Network Europe (PCNE) classification system was used to classify problems (e.g., treatment effectiveness or treatment safety) and causes (e.g., no indication for a drug, drug dose too high or duration or treatment too long) [7]. In this classification system, problems with treatment effectiveness were defined as a (potential) problem with the (lack of) effect of the pharmacotherapy, and problems with treatment safety were defined as the suffering of a patient from an adverse drug event. Anatomical therapeutic chemical (ATC) codes given by the World Health Organization (WHO) Collaboration Centre for drug statistics methodology were used to define drug classes [20].

The acceptance rate of recommendations among clinical physicians, GPs and elderly care specialists were determined. The acceptance rate was defined as the proportion of patients for whom at least one of the recommendations was accepted by the treating physician. A distinction was made between acceptance rate of clinical physicians and GPs or elderly care specialists. For patients who were admitted following the ED visit, the electronic patient management system was used as a source to determine acceptance rate. For patients discharged from the ED, the discharge letter and follow-up report were used to determine acceptance rate.

### Outcome measures

The primary objective in this study was to measure the prevalence and type of DRPs among a group of older patients living with frailty visiting the ED by performing medication reviews. The second objective was to determine the acceptance rate of recommendations proposed by the clinical pharmacist among clinical physicians and GPs and elderly care specialists.

### Statistical analyses

Data were analyzed using R, version 4.0.0. Categorical variables were expressed as counts with corresponding percentages, and continuous data were expressed as mean and standard deviation. The prevalence of DRPs and acceptance rate were reported using descriptive statistics.

## Results

### Patients

We included 356 ED visits of older people living with frailty in our study. Demographic and drug-related patient characteristics are shown in Table 2. Data are presented for the total population, and for patients with or without DRPs separately. 22 Patients were admitted to the ED twice during the study period and both admissions were included in data analyses. Interventions were recommended for 76% of ED visits. The majority of patients were admitted to the hospital after presentation at the ED (*n* = 233, 66%). A fracture or fall was the main reason for ED visits (*n* = 192, 54%).

**Table 2:** Demographic and drug-related patient characteristics (*n* = 356). Values are reported in number (n) and %, unless otherwise specified

Variable	n (%)	≥ 1 DRP n (%)	0 DRPs n (%)
Emergency department visits	356 (100)	270 (76)	86 (24)
Age, mean (SD)	83 (6.8)	83 (6.8)	82 (6.5)
Sex			
Male	139 (39)	105 (39)	34 (40)
Female	217 (61)	165 (61)	52 (60)
Number of drugs per patient, mean (SD)	8 (4.7)	9 (4.5)	5 (4.0)
Polypharmacy	158 (45)	127 (47)	31 (36)
Cause of emergency department visit			
Bleeding	14 (3.9)	13 (4.8)	1 (1.2)
Vomit/diarrhea	7 (2.0)	6 (2.2)	1 (1.2)
Collapse/hypotension/vertigo	9 (2.5)	9 (3.3)	0
Delirium/confused/drowsy	11(3.1)	11 (4.1)	0
Electrolyte disturbances/dehydration	7 (2.0)	6 (2.2)	1 (1.2)
Fracture/fall	192 (54)	142 (53)	50 (58)
Heart failure	1 (0.3)	0 (0)	1 (1.2)
Kidney failure	1 (0.3)	1 (0.4)	0
Obstipation/ileus	2 (0.6)	2 (0.7)	0
Disrupted blood glucose levels	2 (0.6)	2 (0.7)	0
Other	110 (31)	78 (29)	32 (37)
Hospital admission			
Yes	233 (66)	181 (67)	52 (60)
No	123 (35)	89 (33)	34 (40)
Number of DRPs	1.5 (1.3)	2.0 (1.1)	NA
No DRP	86 (24)	NA	NA
1 DRP	104 (29)	104 (38)	NA
2 DRPs	95 (27)	95 (36)	NA
≥ 3 DRPs	71 (20)	71 (26)	NA

*Abbreviations*: *DRP* drug related problem, *NA* not applicable, *SD* standard deviation

### Prevalence of DRPs

A total of 548 DRPs were identified, with a mean (standard deviation, SD) of 1.5 (1.3) DRPs per patient. In 76% of patients at least one DRP was identified. The mean time for performing a medication review was 10 min per patient. The majority of included patients had at least 1 DRP. The most common problem was treatment safety (44%), followed by treatment effectiveness (31%), and unnecessary treatment (20%). Causes of DRPs are presented in Table 3. From the total of 548 DRPs, the most common causes were inappropriate drug selection (72%), and dose selection (18%). Within the group of inappropriate drug selection, the most common cause was the absence of an indication (25%), followed by no treatment for the indication (14%), and drug selection according to the guidelines but contra-indicated (13%). In the group of inappropriate drug selection, the majority of DRPs were caused by drug doses being too high (9.3%), and by too frequent dosing regimens (4.6%).

**Table 3:** Causes of DRPs according to the Pharmaceutical Care Network Europe (PCNE) classification system. In total, 548 DRPs were identified during the study period

Cause	n (%)
*C1: Drug selection*	394 (72)
C1.1 Inappropriate drug according to guidelines	62 (11)
C1.2 According to guidelines but contra-indicated	71 (13)
C1.3 No indication for drug	139 (25)
C1.4 Inappropriate combination of drug, or drugs and food	2 (0.4)
C1.5 Inappropriate duplication of therapeutic group or active ingredient	10 (1.8)
C1.6 No treatment for indication	75 (14)
C1.7 Too many drugs for indication	35 (6.4)
*C2: Drug form*	0 (0)
C2.1 Inappropriate drug form	0 (0)
*C3: Dose selection*	97 (18)
C3.1 Drug dose too low	12 (2.2)
C3.2 Drug dose too high	51 (9.3)
C3.3 Dose not frequent enough	5 (0.9)
C3.4 Dose too frequent	25 (4.6)
C3.5 Dosing time is wrong, unclear or missing	4 (0.7)
*C4: Treatment duration*	31 (5.7)
C4.1 Duration of treatment too short	0 (0)
C4.2 Duration of treatment too long	31 (5.7)
*C5: Dispensing*	0 (0)
*C6: Drug use*	0 (0)
*C7: Patient related*	5 (0.9)
C7.1 Patient takes less or none of the drug	3 (0.5)
C7.8 Patient administered/uses drug in a wrong way	2 (0.4)
*C8: Other causes*^*a*^	21 (3.8)
C8.1 No or inappropriate outcome monitoring (TDM)	7 (1.3)
C8.2 Other cause	14 (2.6)

Abbreviation: *TDM* therapeutic drug monitoring

^**a**^This category includes, among other causes, considering a fixed dose combination and allergy registration in the electronic patient management system

### Drug classes of DRPs

Different drug classes were involved in DRPs. In total, DRPs were most common in ATC group A (alimentary tract and metabolism; 144 DRPs), group C (cardiovascular system; 126 DRPs), and group N (nervous system; 107 DRPs). The distribution DRP causes per ATC group are presented in Table 4.

**Table 4:** Distribution of causes of drug-related problems (DRPs) per anatomical therapeutic chemical (ATC) group. Numbers in bold are the highest values in each column

	**Drug selection (C1)**					**Dose selection (C3)**	**Treatment duration (C4)**	**Patient related (C7)**	**Other causes (C8) **^**a**^
	Inappropriate drug according to guidelines (C1.1)	Contra-indicated (C1.2)	No indication for drug (C1.3)	No treatment for indication (C1.6)	Too many drugs for indication (C1.7)				
	*n* = 62	*n* = 71	*n* = 139	*n* = 75	*n* = 35	*n* = 97	*n* = 31	*n* = 5	*n* = 21
**ATC group**									
Alimentary tract and metabolism (ATC group A)	3	6	**82**	**28**		22	1	1	1
Blood and blood-forming organs (ATC group B)	1	4	8	6		11	7		1
Cardiovascular system (ATC group C)	6	**35**	25	3	**32**	17		1	7
Genitourinary system and reproductive hormones (ATC group G)		2					**13**		1
Systemic hormonal preparations excluding reproductive hormones and insulin (ATC group H)		1	1	5					1
Anti-infective agents for systemic use (ATC group J)	1	2	2	1		2	2		
Antineoplastic and immunomodulating agents (ATC group L)		1	2				1		1
Musculoskeletal system (ATC group M)	3	11	2	20		1	2		
Nervous system (ATC group N)	**41**	8	9	8	1	**33**	1		6
Respiratory system (ATC group R)	3		5	2		7		2	
Various (ATC group V)	4	1	3	2	2	4	4	1	3

^**a**^This category includes, among other causes, the absence of therapeutic drug monitoring or the use of controlled-release tablets instead of immediate release tablets

### Acceptance rates

After ED visit, 169 patients with at least 1 DRP were admitted to the hospital. Recommended interventions were accepted by the clinical physician for 93 patients (55%) and recommendations were documented in the discharge letter to primary care for 61 patients (36%) upon discharge.

Of 86 patients being discharged directly from the ED with at least one DRP, recommended interventions were included in the discharge letter to the GP or elderly care specialist for 47 patients (55%). The acceptance rate of these recommendations was 32% (15 out of 47). Five patients passed away during the follow-up period.

## Discussion

In this study, 548 DRPs were identified in older patients living with frailty visiting the ED with a mean of 1.5 DRP per patient. The majority of DPRs were caused by inappropriate drug selection (72%) and dose selection (18%). The acceptance rate or recommended interventions among clinical physicians was 55% compared to 32% among GPs and elderly care specialists.

In this study, the prevalence of patients with at least 1 DRP was 76%. Similar percentages have been described in literature. In older patients admitted to the medical and surgical wards, DRPs were identified in 82% of patients [21, 22]. Furthermore, the mean number of DRPs per patient was comparable [22]. These results show that by practical implementation of a medication review program at the ED, comparable DRP rates are found as in literature. In a recently published meta-analysis, the impact of pharmacist interventions in the ED were studied [12]. Overall, by pharmacist interventions the percentage of patients with at least one medication error was reduced by 73% and the mean number of medication errors per patient was reduced by 0.33. In this meta-analysis, 7 studies were performed exclusively in older patients (aged 65 years or older). The benefit of pharmacist interventions was similar in older patients and patients younger than 65 years old. In our study, we included older patients living with frailty, which is a population we believe might benefit more from pharmacist-led medication reviews at the ED.

The acceptance rate of recommendations among GPs for older patients at the ED in clinical trials ranges from 27 to 66% [10, 23]. The acceptance rate of DRPs among clinical physicians for admitted patients with at least one recommendation varies from 55 to 82% [22, 24, 25]. Our study observed a low acceptance rate of DRPs among GPs and elderly care specialists (32%). This low rate of acceptance may have several potential causes. Firstly, GPs received DRPs via discharge letters, however, we found that only 55% of the recommendations were included in these letters, suggesting that nearly half of the DRPs were not brought to the attention of the GPs or elderly care specialists. Furthermore, it is known that recommendations are more often accepted when transmitted orally rather than by software [25]. Second, GPs and elderly care specialists were not informed about the geriatric assessment including a medication review, whereas clinical physicians were informed. Lastly, GPs may know of treatment indications that are unknown in the clinical setting, for which certain drugs need to be continued.

A strength of this study is the integration of a pharmacist as a member of the geriatric care team. Additionally, a widely adopted classification system was used to categorize DRPs. Another strength of this study was the determination of the acceptance rate in both admitted patients and those being discharged from the ED, which is an aspect not commonly addressed in other studies. Finally, performing medication review was incorporated as part of routine patient care.

This study also has some limitations. Firstly, the effect of medication reviews on clinical outcomes in the ED has not been assessed. Additionally, the clinical significance of the recommendations is uncertain, as we did not evaluate the impact on quality of life or patient well-being. Moreover, it is not known if the recommendations were still implemented after the follow-up period. Lastly, the retrospective nature of the study may result in incomplete data on the acceptance rate, as the treating physician may not have documented all changes in medication and considerations thoroughly.

The practical implications of the study's findings suggest that prior to implementing a medication review program in the ED, healthcare networks should be informed of the program's existence. This may result in increased support for the recommendations, leading to a potential increase in the rate of acceptance. The positive outcomes were largely due to the close collaboration with the geriatric department, which led to higher acceptance rates among admitted patients. Based on our experience, conducting medication reviews can be seamlessly integrated into routine care and performed efficiently by a team of pharmacists.

## Conclusion

Pharmacist-led medication reviews as part of a geriatric care team identified at least one DRPs in 76% of older patients living with frailty at the ED. The acceptance rate among clinical physicians in admitted patients was higher (55%) than the acceptance rate among GPs or elderly care specialists in discharged patients (32%).

## Supplementary Information

**Additional file 1.**

## Data Availability

The datasets used and/or analyzed during the current study are available from the corresponding author on reasonable request.

## Abbreviations

ANP: Advanced nurse practitioner
ATC: Anatomical therapeutic chemical
DRP: Drug related problem
ED: Emergency department
PCNE: The Pharmaceutical Care Network Europe
SD: Standard deviation
START STOPP criteria: Screening Tool to Alert to Right Treatment and Screening Tool of Older Persons' Prescriptions criteria
WHO: World Health Organization
