# Supplementary material for: Assessment of drug-related problems at the emergency department in older patients living with frailty: pharmacist-led medication reviews within a geriatric care team
Source: BMC Geriatr. 2023 Apr 5;23:215. doi: 10.1186/s12877-023-03942-x (PMC10074685; doi:10.1186/s12877-023-03942-x)
Supplement: Supplementary file 1 — Additional file 1. [file 12877_2023_3942_MOESM1_ESM.docx]

**Supplementary files**

**Supplementary Table 1.** Trigger tool to identify adverse events [11].

| **Trigger** | **Potentially associated drug** |
| --- | --- |
| Fracture/fall | Psychoactive drugs/corticosteroids/antihypertensive drugs |
| Collapse/hypotension/dizziness | Antihypertensive drugs/anti-arrhythmic drugs/ Psychoactive drugs |
| Bleeding/increased INR | Anticoagulants/thrombocyte aggregation inhibitors/NSAIDs |
| Electrolyte imbalance/dehydration | Diuretics/ACE-inhibitors/AII-inhibitors/NSAIDs/antidepressants drugs |
| Renal insufficiency | ACE-inhibitors/AII-inhibitors/NSAIDs |
| Blood sugar imbalance | Glucose-lowering drugs/ corticosteroids |
| Heart failure | NSAIDs |
| Obstipation/ileus | Opioids/calcium channel blockers |
| Vomiting/diarrhea | Antibiotics |
| Delirium/ drowsiness | Psychoactive drugs//antihypertensive drugs/ anti-arrhythmic drugs/alpha blockers/ benzodiazepines |

Abbreviations: ACE = angiotensine converting enzyme, AII = angiotensine II, INR = international normalized ratio, NSAIDs = non-steroidal anti-inflammatory drugs
